# Impact of pathological tumor stage for salvage radiotherapy after radical prostatectomy in patients with prostate-specific antigen < 1.0 ng/ml

**DOI:** 10.1186/1748-717X-6-150

**Published:** 2011-11-05

**Authors:** Rei Umezawa, Hisanori Ariga, Yoshihiro Ogawa, Keiichi Jingu, Haruo Matsushita, Ken Takeda, Keisuke Fujimoto, Toru Sakayauchi, Toshiyuki Sugawara, Masaki Kubozono, Kakutaro Narazaki, Eiji Shimizu, Yoshihiro Takai, Shogo Yamada

**Affiliations:** 1 Department of Radiation Oncology, Tohoku University School of Medicine, Seiryou-machi 1-1, Aobaku, Sendai, Japan

**Keywords:** Prostate cancer, Radiotherapy, Radical Prostatectomy, PSA, Pathological tumor stage

## Abstract

**Background:**

To evaluate prognostic factors in salvage radiotherapy (RT) for patients with pre-RT prostate-specific antigen (PSA) < 1.0 ng/ml.

**Methods:**

Between January 2000 and December 2009, 102 patients underwent salvage RT for biochemical failure after radical prostatectomy (RP). Re-failure of PSA after salvage RT was defined as a serum PSA value of 0.2 ng/ml or more above the postradiotherapy nadir followed by another higher value, a continued rise in serum PSA despite salvage RT, or initiation of systemic therapy after completion of salvage RT. Biochemical relapse-free survival (bRFS) was estimated using the Kaplan-Meier method. Multivariate analysis was performed using the Cox proportional hazards regression model.

**Results:**

The median follow-up period was 44 months (range, 11-103 months). Forty-three patients experienced PSA re-failure after salvage RT. The 4-year bRFS was 50.9% (95% confidence interval [95% CI]: 39.4-62.5%). In the log-rank test, pT3-4 (p < 0.001) and preoperative PSA (p = 0.037) were selected as significant factors. In multivariate analysis, only pT3-4 was a prognostic factor (hazard ratio: 3.512 [95% CI: 1.535-8.037], p = 0.001). The 4-year bRFS rates for pT1-2 and pT3-4 were 79.2% (95% CI: 66.0-92.3%) and 31.7% (95% CI: 17.0-46.4%), respectively.

**Conclusions:**

In patients who have received salvage RT after RP with PSA < 1.0 ng/ml, pT stage and preoperative PSA were prognostic factors of bRFS. In particular, pT3-4 had a high risk for biochemical recurrence after salvage RT.

## Background

Radical prostatectomy (RP) is one of the curative treatments for prostate cancer. However, biochemical recurrence after radical prostatectomy occurs in approximately 15% to 40% of patients within 5 years [1,2]. Approximately one third of patients with biochemical recurrence will have distant metastases, and the median actuarial period to development of metastases following prostate-specific antigen (PSA) elevation is 8 years [3]. Many studies have demonstrated that salvage radiotherapy (RT) for biochemical recurrence after RP is effective and enables long-term suppression of PSA elevation [4]. Trock et al. reported that 5- and 10-year prostate cancer-specific survival rates were 88% and 62%, respectively, for patients with no salvage treatment and 96% and 86%, respectively, for patients who received salvage RT alone [5]. Recent studies have suggested that early RT is more effective than delayed RT. Some studies have demonstrated that pre-RT PSA is a prognostic factor [4,6-14]. Based on results of those studies, it seems that pre-RT PSA < 1.0 ng/ml as a cutoff value is a factor predicting PSA re-failure after salvage RT [4,7,8,12], though according to a consensus panel report published by the American Society of Therapeutic Radiology and Oncology (ASTRO), early treatment (PSA < 1.5 ng/ml) is more successful than later treatment [15]. However, even some patients with pre-RT PSA < 1.0 ng/ml who have received salvage RT have biochemical recurrence. The objective of this study was to evaluate prognostic factors in salvage RT after RP for patients with pre-RT PSA < 1.0 ng/ml.

## Methods

### Patients

Between January 2000 and December 2009, 102 patients received salvage RT for biochemical failure after RP in Tohoku University Hospital and seven affiliated hospitals. Although the American Urological Association (AUA) defines biochemical recurrence following RP as initial serum PSA of ≥ 0.2 ng/ml with a second confirmatory level of > 0.2 ng/ml [16], the main criterion for salvage RT in this study was that PSA after RP was 0.1 ng/ml or more or that PSA after RP was three consecutive increasing. Patients with massive local recurrence that was detectable by CT or MRI or patients with lymph node or distant metastasis were excluded from this study. Patients who continued to receive hormone therapy for PSA failure after RP but became resistant to the hormone therapy were also excluded.

### PSA doubling time

PSA doubling time (PSADT) was calculated using PSA values above 0.1 ng/ml after RP until the start of salvage RT. PSADT was not calculated for patients who did not have PSA above 0.1 ng/ml. PSADT was estimated by the natural log of 2 (0.693) divided by the slope of the linear regression line of PSA over time [3].

### Radiotherapy

The prostate bed, the bladder neck, the urethral anastomosis and the seminal vesicle bed (in the case of invasion to seminal vesicle) were defined as the clinical target volume (CTV) with references to preoperative computed tomography or magnetic resonance imaging. The planning target volume (PTV) included the CTV with a margin of approximately 1 cm in all directions. The leaf margin was 0.5 cm in all directions. Salvage RT was delivered using photon beams of 10 MV or 15 MV mostly with the four-field technique, three-dimensional conformal RT. The median RT dose was 64 Gy (range, 60-72 Gy). The numbers of patients receiving 60 Gy at 2 Gy daily, 62 Gy at 2 Gy daily, 64 Gy at 2 Gy daily, 64.8 Gy at 1.8 Gy daily, 70 Gy at 2 Gy daily and 72 Gy at 1.2 Gy per fraction twice daily (hyperfractionation) to the prostate bed were 18, 1, 67, 12, 3 and 1, respectively. The RT dose was prescribed at the center of the PTV. None of the patients underwent whole pelvic irradiation.

### Follow-up

Re-failure of PSA after salvage RT was defined as a serum PSA value of 0.2 ng/ml or more above the postradiotherapy nadir followed by another higher value, a continued rise in serum PSA despite salvage RT, or initiation of systemic therapy after completion of salvage RT [17]. The time to PSA re-failure after salvage RT was calculated from the first day of RT. Measurement of PSA after salvage RT was done at least once every 3 months.

### Statistical analysis

Biochemical relapse-free survival (bRFS) was estimated using the Kaplan-Meier method, and the log-rank test was used to analyze differences between patient subgroups categorized by prognostic variables. Multivariate analysis was performed using the Cox proportional hazards regression model. Hazard ratios are presented for each prognostic factor. We evaluated pathologic tumor (pT) stage, surgical margin, Gleason score (GS), preoperative PSA, pre-RT PSA (Pre-RT PSA of patients who received hormone therapy was evaluated as that before hormone therapy.), PSADT, dose to the prostate bed, biological effective dose (BED) (calculated using α/β = 1.5 according to the LQ model) [18], time from RP to the start of RT and hormonal therapy as prognostic factors. Multivariate analysis included factors with p < 0.10 in univariate analysis. All tests were two-sided, and statistical significance was set at the level of p < 0.05. Statistical analysis was performed using the Statistical Package for Social Sciences (SPSS) version 14.0 (SPSS, Chicago, IL).

### Toxicity

Complications due to salvage RT were evaluated according to the common terminology criteria for adverse events (CTCAE) ver.4.0. Late toxicity was defined as a complication occurring more than three months after salvage RT.

### Ethics

Written informed consent for treatment was obtained from all patients, and this retrospective study was performed according to the principles of the Declaration of Helsinki (2008).

## Results

Patient and tumor characteristics are shown in Table 1. Median age at salvage RT was 67 years (range, 49-81 years). Median pre-RT PSA was 0.240 (range, 0.011-0.994 ng/ml). Median preoperative PSA was 8.8 ng/ml (range, 1.6-120 ng/ml). Median PSADT was 6.83 months (range, 0.58-41.9 months).

**Table 1:** Patients' characteristics

Characteristic	**No**.	Median (range)
Age at salvage RT (years)		67 (49-81)
Pathologic tumor stage		
T1	2	
T2	41	
T3	57	
T4	1	
Unknown	2	
Surgical margin		
Positive	48	
Negative	51	
Unknown	3	
Gleason score		
≤ 6	31	
7	35	
8 ≥	31	
Unknown	5	
Preoperative PSA (ng/ml)		8.8 (1.6-120)
< 10	58	
≥ 10	44	
Pre-RT PSA (ng/ml)		0.240 (0.011-0.994)
< 0.25	52	
≥ 0.25	50	
PSA doubling time (months)		6.83 (0.58-41.9)
< 7	43	
≥ 7	43	
Not available	16	
RT dose (Gy) (BED, α/β = 1.5)		64 (149.3) (60-72 [129.6-163.3])
60 at 2 daily (140.0)	18	
62 at 2 daily (144.7)	1	
64 at 2 daily (149.3)	67	
64.8 at 1.8 daily (142.6)	12	
70 at 2 daily (163.3)	3	
72 at 1.2 per fraction twice daily (HF) (129.6)	1	
Time from RP to RT (months)		21 (5-99)
< 24	56	
≥ 24	46	
Hormone therapy		
Done	29	
Not done	73	

Abbreviations: RT = radiotherapy, PSA = prostate-specific antigen, BED = biological effective dose, HF = hyperfractionation, RP = radical prostatectomy

Hormone therapy was given to 29 patients before and/or during salvage RT for a median period of 6 months (range, 1-18 months). Eleven of those patients continued to receive hormone therapy for a median period of 12 months (range, 1-15 months) after salvage RT. Two patients received hormone therapy for PSA failure and stopped the therapy because of decrease in PSA but re-started the therapy when PSA had increased again. No patient received adjuvant hormone therapy after RP.

**Figure 1:**
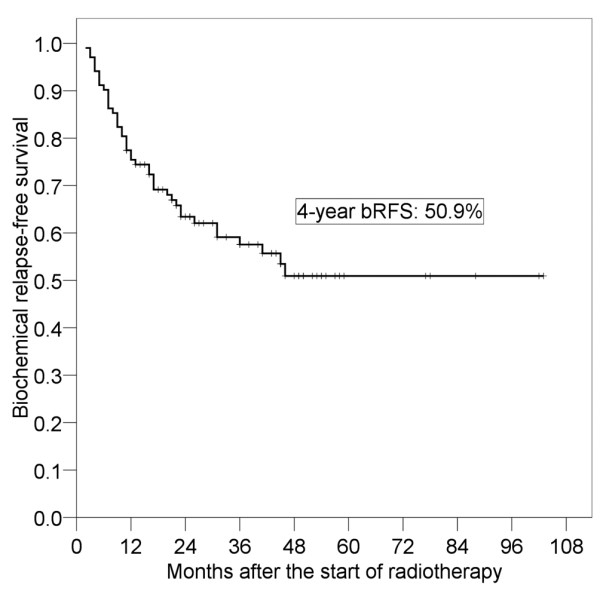
**Biochemical relapse-free survival (bRFS) after salvage radiotherapy**.

The median follow-up period from the start of salvage RT was 44 months (range, 11-103 months). The 4-year overall survival rate was 97.3% (95% confidence interval [95% CI]: 93.6-100%). No patient died of prostate cancer, though one patient died of esophageal cancer and one patient died of bile duct cancer. PSA decreased in 64 of the 73 patients who received salvage RT after RP without hormone therapy. Forty-three patients had re-failure after salvage RT at the last observation date. The 4-year bRFS rate was 50.9% (95% CI: 39.4-62.5%) (Figure 1). Multiple lung metastases occurred in one patient 17 months after salvage RT, and bone metastases occurred in two patients 36 and 43 months after salvage RT.

**Figure 2:**
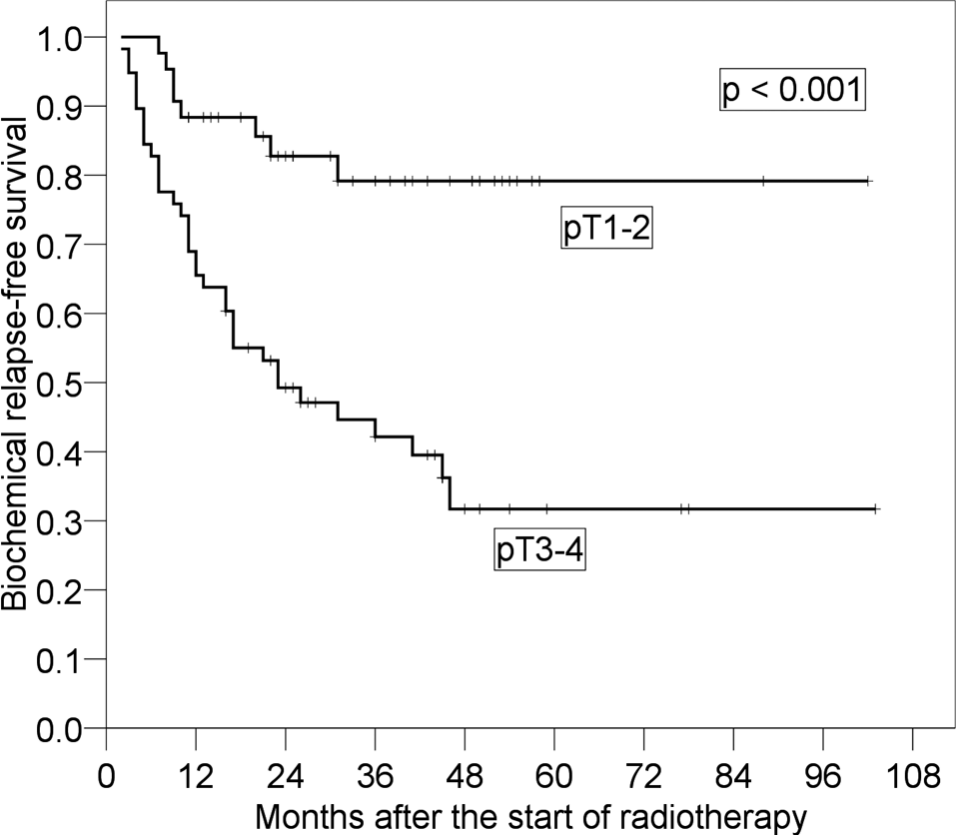
**Four-year biochemical relapse-free survival in patients with pT3-4 and that in patients with pT1-2**.

**Figure 3:**
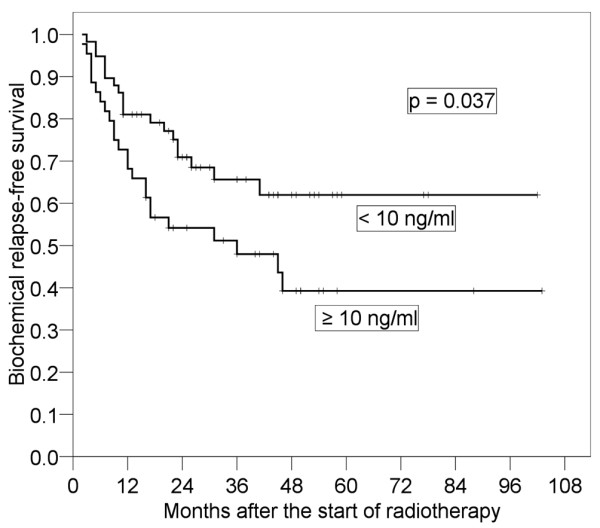
**Four-year biochemical relapse-free survival in patients with preoperative prostate-specific antigen (PSA) ≥ 10 ng/ml and that in patients with preoperative PSA < 10 ng/ml**.

**Figure 4:**
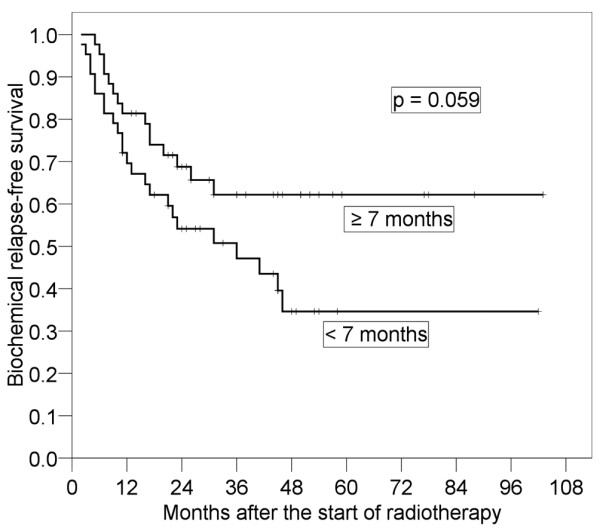
**Four-year biochemical relapse-free survival in patients with prostate specific-antigen doubling time (PSADT) ≥ 7 months and that in patients with PSADT < 7 months**.

**Figure 5:**
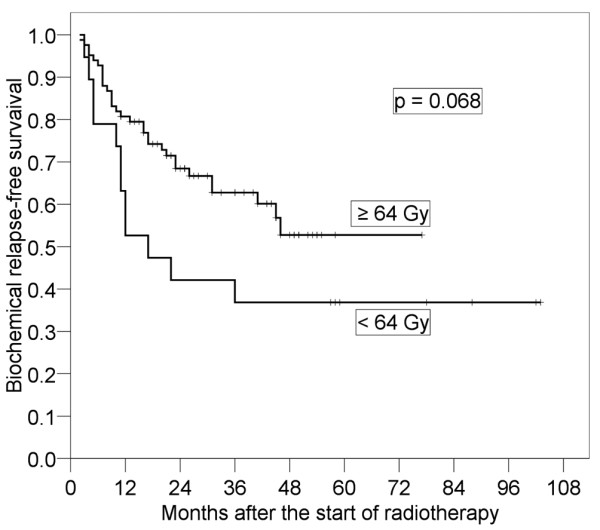
**Four-year biochemical relapse-free survival in patients with radiotherapy (RT) dose ≥ 64 Gy and that in patients with RT dose < 64 Gy**.

Results of the log-rank tests presented in Table 2 show the 4-year bRFS for each prognostic factor before salvage RT. It was found that pT stage (p < 0.001) and preoperative PSA (p = 0.037) were significant prognostic factors. The 4-year bRFS for pT1-2 and pT3-4 were 79.2% (95% CI: 66.0-92.3%) and 31.7% (95% CI: 17.0-46.4%), respectively (Figure 2). We also analyzed bRFS for extracapsular extension and seminal vesicle invasion. The 4-year bRFS for positive and negative extracapsular extension were 79.9% (95% CI: 65.4-94.5%) and 35.9% (95% CI: 21.0-49.8%) (p = 0.003), respectively. The 4-year bRFS for positive and negative seminal vesicle invasion were 56.9% (95% CI: 44.3-71.0%) and 19.0% (95% CI: 0-39.1%) (p = 0.004), respectively. The 4-year bRFS for preoperative PSA < 10 ng/ml and ≥ 10 ng/ml were 62.0% (95% CI: 47.7-76.3%) and 39.3% (95% CI: 22.5-56.0%), respectively (Figure 3). Although not significant, PSADT (p = 0.059) and RT dose (p = 0.068) tended to be prognostic factors. The 4-year bRFS for PSADT < 7 months and ≥ 7 months were 34.6% (95% CI: 17.5-51.8%) and 62.2% (95% CI: 46.7-77.7%), respectively (Figure 4). The 4-year bRFS for dose < 64 Gy and ≥ 64 Gy were 36.8% (95% CI: 15.2-58.5%) and 52.7% (95% CI: 39.5-69.1%), respectively (Figure 5). When we evaluated bRFS in 73 patients without hormone therapy, pT stage (p < 0.001) and preoperative PSA (p = 0.018) were also significant prognostic factors. In those patients, the 4-year bRFS for pT1-2 and pT3-4 were 78.5% (95% CI: 62.9-94.2%) and 29.6% (95% CI: 13.7-45.6%), respectively, and the 4-year bRFS for preoperative PSA < 10 ng/ml and ≥ 10 ng/ml were 64.0% (95% CI: 48.5-79.5%) and 33.9% (95% CI: 14.3-53.6%), respectively.

**Table 2:** Results of log-rank tests

Factor	4-year bRFS (%) (95% CI)	p value
Pathologic tumor stage		< 0.001
T1-2	79.2 (66.0-92.3)	
T3-4	31.7 (17.0-46.4)	
Surgical margin		0.652
Positive	48.6 (32.0-65.1)	
Negative	57.1 (42.7-71.4)	
Gleason score		0.189
≤ 7	58.9 (44.5-73.3)	
≥ 8	35.2 (14.7-51.5)	
Preoperative PSA (ng/ml)		0.037
< 10	62.0 (47.7-76.3)	
≥ 10	39.3 (22.5-56.0)	
Pre-RT PSA (ng/ml)		0.620
< 0.25	54.5 (37.5-71.6)	
≥ 0.25	45.9 (30.2-64.9)	
PSA doubling time (months)		0.059
< 7	34.6 (17.5-51.8)	
≥ 7	62.2 (46.7-77.7)	
RT dose (Gy)		0.068
< 64	36.8 (15.2-58.5)	
≥ 64	51.7 (37.6-65.9)	
BED (Gy) (α/β = 1.5)		0.213
< 145	41.4 (23.5-59.3)	
≥ 145	54.3 (39.5-69.1)	
Time from RP to RT (months)		0.310
< 24	45.2 (29.1-61.4)	
≥ 24	57.4 (40.9-73.9)	
Hormone therapy		0.627
Done	50.5 (20.2-72.9)	
Not done	50.7 (37.6-63.7)	

Abbreviations: bRFS = biochemical relapse-free survival, RT = radiotherapy, PSA = prostate-specific antigen, BED = biological effective dose, RP = radical prostatectomy, CI = confidence interval

**Figure 6:**
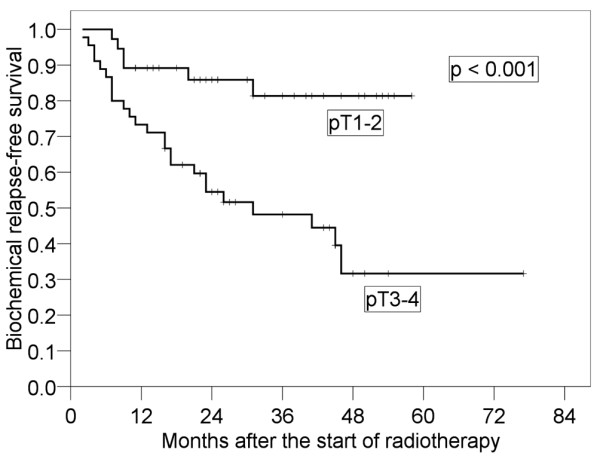
**Four-year biochemical relapse-free survival for pT3-4 and that for pT1-2 in patients who received 64 or 64.8 Gy to the prostate bed**.

In 79 patients who received 64 or 64.8 Gy to the prostate bed, the 4-year bRFS for pT1-2 and pT3-4 were 85.5% (95% CI: 73.6-97.3%) and 28.8% (95% CI: 7.87-49.8%) (p < 0.001), respectively (Figure 6).

Multivariate analysis was evaluated by pT stage, preoperative PSA, PSADT and RT dose. Only pT stage was a significant prognostic factor (p = 0.003) and the hazard ratio (HR) with pT3-4 was 3.512 (95% CI: 1.535-8.037) (Table 3). After adjusting for pT, none of the other variables retained prognostic significance.

**Table 3:** Multivariate analysis of parameters with bRFS

Factors	Hazards ratio (95% CI)	p value
Pathologic tumor stage		0.003
T1-2	Reference	
T3-4	3.512 (1.535-8.037)	
Preoperative PSA (ng/ml)		0.177
< 10	Reference	
≥ 10	1.566 (0.816-3.006)	
PSADT (months)		0.272
< 7	Reference	
≥ 7	0.686 (0.350-1.344)	
RT dose (Gy)		0.357
< 64	Reference	
≥ 64	0.712 (0.345-1.469)	

Abbreviations: bRFS = biochemical relapse-free survival, PSA = prostate-specific antigen, PSADT = prostate-specific antigen doubling time, RT = radiotherapy, RP = radical prostatectomy, CI = confidence interval

Grade 2 late urinary tract complication was observed in one patient who suffered from urinary occlusion. Grade 3 late rectal complication was observed in one patient who suffered from rectal bleeding.

## Discussion

Moreira et al. reported that the 1-, 3- and 5-year risks of receiving any salvage treatment after RP were 29%, 48% and 53%, respectively [19]. Salvage RT is one of the major treatments for biochemical recurrence after RP, and many studies have shown that salvage RT is effective [4,6-14,20-27]. In our study, PSA decreased in 64 of the 73 patients who received salvage RT after RP without hormone therapy, suggesting that this treatment was effective for biochemical failure after RP. Those patients might have had at least a component local disease. PSA did not decrease in the patient who had multiple lung metastases. Although comparison with results of previous studies might not be appropriate differences in race, conditions under which salvage RT was performed and criteria of re-failure after salvage RT, bRFS in the present study was similar to that in previous studies (Table 4). However, significant prognostic factors for re-failure of salvage RT in those studies were different. Some of those studies showed that pre-RT PSA < 1.0 ng/ml was a prognostic factor [4,7,8,12]. In those studies, median values of pre-RT PSA were higher (0.7-4.5 ng/ml) than that in the present study (0.240 ng/ml). However, even some patients with pre-RT PSA < 1.0 ng/ml who have received salvage RT experience biochemical recurrence. Therefore, we retrospectively evaluated prognostic factors in salvage RT for patients with pre-RT PSA < 1.0 ng/ml.

**Table 4:** Past studies on salvage RT for biochemical recurrence after RP

Study	Patients (n)	Median pre-RT PSA (ng/ml)	prognostic factors after salvage RT	bRDS(%)
Hagan et al [7]	91	4.5	pre-RT PSA (< 1.0 ng/ml)	55 (5-year)
Quero et al [8]	59	1.43	pre-RT PSA (< 1.0 ng/ml)	41.2 (5-year)
Macdonald et al [9]	121	1.4	pre-RT PSA (< 0.2 ng/ml)	NA
Anscher et al [10]	89	1.4	pre-RT PSA (< 2.5 ng/ml), RT dose (> 65 Gy)	50 (4-year)
Chawla et al [20]	54	1.3	Gleason score (≤ 6), time to detectable postoperative PSA	35 (5-year)
Tsien et al [21]	57	1.2	Gleason score (< or = 7)	58 (5-year)
Neuhof et al [4]	171	1.1	Gleason score (< 7), pre-RT PSA (< 1.0 ng/ml)	35.1 (5-year)
Jacinto et al [22]	43	0.87	PSADT (> 4.0 months)	71 (3-year)
Taylor et al [23]	66	0.8	delayed rise in PSA after RP	66 (5-year)
Pazona et al [11]	307	0.8	pre-RT PSA (< 1.3 ng/ml), Seminal vesicle	40 (5-year)
Stephenson et al [6]	501	0.72	Gleason score (≤ 7), pre-RT PSA (≤ 2.0 ng/ml), PSADT (> 10 months)	45 (4-year)
Buskirk et al [12]	368	0.7	pT2-3a, Gleason score (≤ 7), Pre-RT PSA (< 1.0 ng/ml)	46 (5-year)
Bernard et al [13]	364	0.6	RT dose (> 66.6 Gy, patients with pre-RT PSA < 0.6 ng/ml)	50 (5-year)
Ward et al [24]	211	0.6	PSADT (> 12 months)	34 (10-year)
King et al [25]	37	0.49	PSAV (≤ 1.0 ng/ml/year)	NA
Wiegel et al [26]	162	0.33	PSA undetectable after salvage RT (< 0.1 ng/ml)	54 (3.5-year)
Tomita et al [27]	51	0.25	PSADT (> 3.0 months), Gleason Score, RT dose (≥ 60 Gy)	55.1 (3-year)
Terai et al [14]	37	0.146	cT1-2, pT2, pre-RT PSA (> 0.15 ng/ml)	54 (5-year)

Abbreviations: RT = radiotherapy, RP = radical prostatectomy, PSA = prostate-specific antigen, bRDS = biochemical relapse-free survival, PSADT = PSA doubling time, PSAV = PSA velocity, NA = not available

In the present study, pre-RT PSA was not a significant factor. Therefore, there might be no need for early salvage RT in patients with pre-RT PSA < 1.0 ng/ml. However, early or more intense salvage RT in cases of high risk for recurrence (e.g., pT3-4 and preoperative PSA > 10 ng/ml) even with PSA < 1.0 ng/ml might be necessary because the rate of biochemical recurrence after salvage RT is relatively high even in patients with pre-RT PSA < 1.0 ng/ml. Further investigation of the prognostic factors of salvage RT other than pre-RT PSA in patients with high risk for recurrence is needed.

Recently, PSADT has been utilized as a prognostic factor for prostatectomy. Pound et al. found that PSADT predicted the likelihood of subsequent development of metastatic disease [3]. Freedland et al. found that prostate cancer-specific survival of patients with PSADT < 9 months (especially < 3 months) was worse than that of patients with PSADT > 15 months [28]. Some past studies have demonstrated that PSADT was also a prognostic factor of salvage RT [6,22,24,27]. Median pre-RT PSA in some of those studies was 1.0 ng/ml or less. In a study by Trock et al. with median pre-RT PSA < 1.0 ng/ml, salvage RT performed within 2 years after biochemical recurrence significantly improved prostate cancer-specific survival among patients with PSADT of less than 6 months (HR: 0.14) [5]. Although not significant in our study, the 4-year bRFS rate of patients with PSADT < 7 months was worse than that of patients with PSADT ≥ 7 months. The reason why PSADT was not a significant factor in our study might be that there were 16 patients for whom PSADT data were not available and the number of cases for analysis was insufficient. If pre-RT PSA is < 1.0 ng/ml, we might be able to refer to PSADT for starting salvage RT. Although PSADT is widely used to predict outcomes such as time to progression, underlying the impressive evidence of predictive value and prognostic value of PSADT are many basic questions about how it should be calculated [29]. Although Arlen et al. demonstrated that PSA kinetics was all calculated from the point of failure of 0.2 ng/ml [30], PSADT was calculated using PSA values above 0.1 ng/ml after RP in our study.

In our study, only pT3-4 was a significant factor predicting PSA re-failure after salvage RT in multivariate analysis. In a study by Wiegel et al., bRFS for pT3-4 was worse than that for pT2 (p = 0.047) [26]. Although bRFS for pT3-4 was poor in patients with PSA < 1.0 ng/ml, there may be some room for improvement in RT for patients with pT3-4. One method for improving bRFS in patients with pT3-4 is postoperative RT. According to the European Organization for Research and Treatment of Cancer (EORTC) 22911, adjuvant external irradiation after radical prostatectomy improves biochemical progression-free survival and local control in patients with positive margins [31]. In that study, the 5-year biochemical progression-free survival rates for patients in the Irradiation group and the Wait and see group with one or more pathological risk factors (capsule perforation, positive surgical margins and invasion of seminal vesicles) were 74.0% and 52.6%, respectively. In the Southwest Oncology Group (SWOG) 8794, adjuvant radiotherapy for patients with pT3 after RP resulted in significant reduction of the risk for PSA relapse (median PSA relapse-free survival: 10.3 years for radiotherapy vs. 3.1 years for observation) [32]. In their further study, metastasis-free survival was significantly greater with adjuvant radiotherapy (93 of 214 patients on the radiotherapy arm vs 114 of 211 patients on the observation arm) and survival improved significantly with adjuvant radiotherapy (88 deaths in 214 patients on the radiotherapy arm vs 110 deaths in 211 patients on the observation arm) [33]. In a study by Jereczek-Fossa et al., failure-free survival of postoperative RT patients was significantly longer than that of patients who had undergone salvage RT (4-year biochemical control rates: 81.7% and 60.5%, respectively) [34]. Postoperative RT was suggested to be more effective than salvage RT in patients with pT3-4. The reason why bRFS for patients with pT3-4 is poor may also be that these patients already have latent lymph node metastases. Spiotto et al. reported that whole pelvic RT resulted in superior bRFS compared with prostate bed RT, especially in high-risk patients with GS ≥ 8, preoperative PSA > 20 ng/ml, pT3, or pathologic lymph node involvement (5-year bRFS: 47% vs 21%) [35]. Whole pelvic RT might also be an effective treatment.

The ASTRO consensus guidelines suggest a minimum of 64 Gy at conventional dose fractionation [15]. In our study, bRFS of patients treated with a dose ≥ 64 Gy tended to be better than that of patients treated with a dose < 64 Gy (51.7% [95% CI: 38.5-67.0%] vs. 36.8% [95% CI: 15.2-58.5%], p = 0.068). At least 64 Gy may be required for salvage RT after RP. King et al. found that salvage RT with 70 Gy was superior to that with 60 Gy (5-year bRFS: 58% vs. 25%) [36]. Bernard et al. found that doses higher than 66.6 Gy resulted in decreased risk of biochemical failure after salvage RT [13]. King et al. reported that the dose-response relationships of salvage RT and definitive external beam radiotherapy for localized prostate cancer were similar [37]. Only in patients treated with 64 or 64.8 Gy, the 4-year bRFS for patients with pT3-4 was much worse than that for patients with pT1-2 (85.5% [95% CI: 73.6-97.3%] and 28.8% [95% CI: 7.87-49.8%], respectively, p < 0.001), (Figure 6). This result suggested that 64 Gy in patients with pT3-4 might be insufficient. The results of a study by Cozzarini et al. provided strong support for the use of RT at doses ≥ 70 Gy in pT3-4 patients [38]. Therefore, patients with pT3-4 might need dose escalation for salvage RT after RP.

There were some limitations in the present study. First, data for patients who received hormone therapy prior to RT may confound the analysis and lessen the significance of some risk factors. The value of PSA is strongly affected by hormone therapy. Therefore, we also evaluated bRFS in 73 patients without hormone therapy. Although the number of samples became small, the result was similar. Second, we analyzed bRFS in the present study retrospectively, and there were two separate definitions of the criterion for salvage RT. The criteria were slightly different between previous studies. AUA defines biochemical recurrence following RP as initial serum PSA of ≥ 0.2 ng/ml with a second confirmatory level of > 0.2 ng/ml [16]. We analyzed bRFS in 65 patients with pre-PSA of ≥ 0.2 ng/ml. The results showed that only pT was only a siginificant prognostic factor (p < 0.001). The bRFS after salvage RT might be affected by pT3-4 after all, even if we consider those limitations.

## Conclusion

Salvage RT is an effective treatment for patients with biochemical recurrence after RP. In patients with PSA < 1.0 ng/ml, pT stage and preoperative PSA were prognostic factors of bRFS. In particular, pT3-4 had a high risk for biochemical recurrence after salvage RT, and more intense treatment is recommended for such patients.

## Competing interests

The authors declare that they have no competing interests.

## Authors' contributions

RU participated in data collection, performed the statistical analysis and drafted the manuscript. HA and YO conceived of the study and helped to draft the manuscript. KJ participated in its design and coordination and helped to draft the manuscript. HM, KT, KF, TS, TS, MK, KN, ES helped with collection of data. YT and SY helped to draft the manuscript. All authors read and approved the final manuscript.
